# Long-Term Young Adult Cancer Survivors with Ovarian Cancer: Subgroup Analysis of the Study “Expression VI–Carolin Meets HANNA–Holistic Analysis of Long-Term Survival with Ovarian Cancer”: The International NOGGO, ENGOT, and GCIG Survey

**DOI:** 10.3390/cancers18071183

**Published:** 2026-04-07

**Authors:** Desislava Dimitrova, Eleftherios Pierre Samartzis, Dario Zocholl, Maria-Pilar Barretina-Ginesta, Katharina Leitner, Pavel Havelka, Patriciu Achimas-Cadariu, Cagatay Taskiran, Suzana Mittelstadt, Els Van Nieuwenhuysen, Gerd Bauerschmitz, Viola Heinzelmann-Schwarz, Ainhoa Madariaga, Uta Ringsdorf, Tibor Zwimpfer, Caterina Madroñal, Hans-Martin Enzinger, Sara Al Rubaish, Jalid Sehouli, Hannah Woopen

**Affiliations:** 1 Department of Gynecology with Center for Oncological Surgery, Charité—Universitätsmedizin Berlin, 13353 Berlin, Germany; desislava.dimitrova@charite.de (D.D.); jalid.sehouli@charite.de (J.S.); 2 North-Eastern German Society for Gynecological Oncology (NOGGO), 13359 Berlin, Germany; 3 Department of Gynecology, University Hospital of Zürich, 8091 Zürich, Switzerland; 4 Swiss GO Trial Group (Swiss GO), 4031 Basel, Switzerland; 5 Institute for Medical Biometry, Informatics and Epidemiology, University Hospital Bonn, University of Bonn, 53127 Bonn, Germany; 6 Medical Oncology Department, Institut Català d’Oncologia, 17007 Girona, Spain; 7 Grupo Español de Investigación en Cáncer de Ovario (GEICO), 28003 Madrid, Spain; ainhoa.madariaga@salud.madrid.org (A.M.);; 8 Department of Gynecology and Obstetrics, Medical University of Innsbruck, 6020 Innsbruck, Austria; 9 Arbeitsgemeinschaft Gynaekologische Onkologie Austria (AGO Austria), 6020 Innsbruck, Austria; 10 Tomas Bata Hospital Zlin, 760 01 Zlín, Czech Republic; 11 Central and Eastern European Gynecologic Oncology Group (CEEGOG), 120 00 Prague, Czech Republic; 12 Surgical Oncology and Gynecologic Oncology, “Iuliu Hațieganu” University of Medicine and Pharmacy—Cluj-Napoca, 400012 Cluj-Napoca, Romania; 13 Surgical Oncology, The Oncology Institute Prof. Dr. I. Chiricuta Cluj-Napoca, 400015 Cluj-Napoca, Romania; 14 VKV American Hospital, 34365 Sisli, Turkey; 15 Turkish Society of Gynecologic Oncology (TRSGO), 06450 Ankara, Turkey; 16 Department of Women’s Health, University Hospital Tübingen, 72076 Tübingen, Germany; 17 DIAK Klinikum Landkreis Schwäbisch Hall, 74523 Schwäbisch Hall, Germany; 18 University Hospitals Leuven, 3000 Leuven, Belgium; 19 Belgium and Luxembourg Gynaecological Oncology Group (BGOG), 3000 Leuven, Belgium; 20 Department of Gynecology and Obstetrics, Universitätsklinikum Göttingen, 37075 Göttingen, Germany; 21 Gynecological Cancer Centre, University Hospital Basel, 4031 Basel, Switzerland; 22 Ovarian Cancer Research, Department of Biomedicine, University of Basel, 4031 Basel, Switzerland; 23 Hospital Universitario 12 de Octubre, 28026 Madrid, Spain; 24 Lahn-Dill Kliniken Wetzlar, 35578 Wetzlar, Germany; 25 Institut d’Oncologia Clínica, 08017 Barcelona, Spain; 26 Sozialstiftung Bamberg–Klinikum Bamberg, 96049 Bamberg, Germany; 27 Dr. Sulaiman Al Habib Medical Group, Alkhober 34423, Saudi Arabia; sararubaish@hotmail.com; 28 Pan-Arabian Research Society of Gynecological Oncology (PARSGO), 13353 Berlin, Germany

**Keywords:** cancer survivorship, ovarian cancer, young adult cancer survivors, long-term side effects

## Abstract

Background/Objectives: Ovarian cancer is typically diagnosed in postmenopausal women, so there are limited data available for young adult cancer survivors (YACS). The aim was to assess the patient perspective of YACS. Methods: In this international and multicenter cross-sectional survey study, patient history, long-term side effects, and patient perspective were assessed. Long-term survival was defined as survival of at least five years after cancer diagnosis. Two groups were defined: (1) 18–40 years and (2) ≥41 years. Results: Altogether, 1833 long-term survivors (LTS) have been recruited, with 1771 patients ≥41 years and 62 patients 18–40 years at recruitment. FIGO stages were similar; among the patients, 99.0% had received primary surgery followed by chemotherapy in 90.3%. Almost 50% still experienced long-term side effects. Patients ≤ 40 years reported more frequently not only gastrointestinal symptoms such as nausea/vomiting (44.4%, *p* = 0.01), bloating (59.3%, *p* = 0.038), and constipation (60%, *p* = 0.015) but also depression (31.4%, *p* = 0.02), lymphedema (45.3%, *p* = 0.026), and concentration difficulties (30.6%, *p* = 0.002). Distress levels were also higher in YACS, especially concerning insurance/finances, work/school, child care, worries, and sadness. Polyneuropathy and secondary cancer were the only side effects that were more frequent in the elder cohort (polyneuropathy: 20.3% vs. 4.3%, *p* = 0.002, and secondary cancer: 8.4% vs. 0%, *p* = 0.014). YACS were more physically active (*p* = 0.003) and interested in studies about long-term cancer survivorship in 87.2%. Conclusions: Long-term side effects are equally common in YACS after ovarian cancer, but with a focus on practical problems, mental health, gastrointestinal problems, and sexuality. This knowledge should be incorporated into follow-up care of ovarian cancer patients in order to improve quality of life.

## Simple Summary

The aim of the current study is to summarize treatment-related symptoms, experiences and specific needs of young adult cancer survivors (YACS) with ovarian cancer. The international survey “Expression VI–Carolin meets HANNA–Holistics Analysis of Long-Term Survival with Ovarian Cancer” included 1833 participants from 14 countries. A 68-item questionnaire assessed information about patients’ medical history, patients’ perspectives on current symptoms, side effects during treatment, long-term toxicities, follow-up care, and health competence. Our analyses show that long-term side effects are very common and that YACS suffer more frequently from gastrointestinal symptoms, distress, and practical problems. Through analyzing and describing the long-term symptoms and treatment-related health issues of YACS with ovarian cancer, we are able to provide organ-specific recommendations for the improvement and adjustment of cancer surveillance. The study results underline the need for multidisciplinary follow-up care and tailored long-term survivorship programs for YACS.

## 1. Introduction

The incidence of cancer in young adults has been increasing in recent decades [1,2,3,4]. It is a heterogeneous group of tumor entities affecting different organ systems, with breast cancer as the leading disease among females. The category of young adults includes individuals between the ages of 15 and 39 [5]. With the improvement of screening strategies and treatment options, the chance of survival is above 80% and results in an increasing number of cancer survivors worldwide [6]. In contrast to people affected by cancer, young adults survive years and decades after the diagnosis face challenges not only at the time of diagnosis but also for their future. They have to deal with specific treatment-related problems, such as second malignancies [7] and long-term side effects such as cardiovascular diseases, endocrine dysfunction, fatigue, cognitive impairments, psychological distress, fertility issues, sexual problems, and limitations in work and educational possibilities [8,9,10,11,12,13,14,15].

There are very few specific recommendations dealing with the needs of young cancer survivors, and the majority are based on experience from survivorship programs for childhood cancer survivors and from adult cancer guidelines. Several reviews focused on exploring the literature and providing age-appropriate recommendations tailored to YACS [16,17,18,19,20,21]. With the increasing number of young adult cancer survivors, there is a strong need to adapt survivorship surveillance programs to better address their specific needs. Ovarian cancer is usually a cancer entity that is diagnosed in postmenopausal women. Therefore, there are hardly any data available on young ovarian cancer survivors, which are important for improving patient-tailored medical care for this growing patient cohort.

The international study “Expression VI—Carolin meets HANNA—Holistic Analysis of Long-term Survivors with Ovarian Cancer” is the largest existing patient cohort of long-term survivors with ovarian cancer [22].

The purpose of the present study is to analyze and characterize the subgroup of young long-term ovarian cancer survivors from the Expression VI cohort and to provide organ-based specific recommendations for YACS to improve follow-up care.

## 2. Materials and Methods

The international study “Expression VI–Carolin meets HANNA–Holistics Analysis of Long-Term Survival with Ovarian Cancer” was initiated by the Department of Gynecology with Center for Oncological Surgery at the Charité–Universitätsmedizin Berlin and by the North-Eastern German Society of Gynaecological Oncology (NOGGO e.V., 13359 Berlin, Germany).

The aim was to increase knowledge of the characteristics, long-term side effects, perspectives and expectations of the growing patient cohort of long-term survivors (LTS) after ovarian cancer. Long-term survival was initially defined as survival of eight years after initial cancer diagnosis and was changed to five-year survival after cancer diagnosis according to the definition of long-term survival by the Gynecologic Cancer Intergroup Consensus guideline in July 2020. Long-term survivors of any stage, independent of their current disease status, could participate [23]. All patients had to have histologically confirmed epithelial ovarian cancer to be able to participate.

The survey consists of 68 questions, ranging from single-choice to multiple-choice questions. Both oncological and medical history were obtained. Patients were asked for their health status, experienced and ongoing physical and psychological side effects, and lifestyle and potential lifestyle modifications after cancer diagnosis. Mental health was assessed with the NCCN distress thermometer. Some questions were derived from previous Expression surveys conducted by the NOGGO.

There was a pilot phase where the survey was tested in a small patient cohort (n = 10) for readability and understandability. As the distress thermometer is validated and most of the questions were already tested in a large patient cohort of prior Expression surveys, readability and understandability were confirmed by all patients in the pilot phase. Ethical approval was obtained by the local ethics committees under the lead of Charité–Universitätsmedizin Berlin (AVD-No: EA2/139/16). The Expression VI is anonymous, and it was assumed that if the patients handed back the filled-out questionnaire, they wanted to participate. Therefore, no consent form was needed.

The survey was professionally translated into 15 languages (Arabic, Czech, Dutch, English, French, German, Hungarian, Italian, Polish, Romanian, Slovakian, Slovenian, Spanish, Turkish, and Ukrainian) and proofread by bilingual doctors before it was made available for ENGOT (European Network of Gynaecological Oncology Trial Groups) and GCIG (Gynecologic Cancer Intergroup) member groups. Centers of the following countries participated: Austria (AGO Austria—Arbeitsgemeinschaft Gynaekologische Onkologie Austria), Belgium (BGOG—Belgium and Luxembourg Gynaecological Oncology Group), Czech Republic (CEEGOG—Central and Eastern European Gynecologic Oncology Group), Germany (NOGGO—North-Eastern German Society of Gynaecological Oncology), Hungary (CEEGOG), Poland (CEEGOG), Romania, Saudi Arabia (PARSGO—Pan-Arabian Research Society of Gynecological Oncology), Slovakia (CEEGOG), Slovenia (CEEGOG), Spain (GEICO—Grupo Español de Investigación en Cáncer de Ovario), Switzerland (Swiss GO Trial Group), Turkey (TRSGO—Turkish Society of Gynecologic Oncology) and Ukraine (CEEGOG). Recruitment began in November 2016 and is still ongoing. The first full-text publication with 1044 participants was published in 2023. Until now, 1833 long-term survivors with ovarian cancer have participated in the study. The Expression VI is available at participating centers, which include university hospitals, local hospitals, clinics, and private practices (for both in-hospital and ambulatory patients), and is also distributed to self-help groups and patient conferences by the Ovarian Cancer Foundation and online (www.noggo.de/studien/umfagen.html, accessed on 7 April 2026). Only 56 patients filled out the questionnaire online. All questionnaires that were distributed via self-help groups, patient conferences, and the Ovarian Cancer Foundation had to be collected via participating study centers to ensure that inclusion criteria were met.

The statistical software R (Version 4.5.2) was used for statistical analyses. Most analyses were descriptive, and the study is exploratory. Continuous data are described using median and range, while categorial data are depicted with frequencies and percentages. The Chi-squared test was used for statistical comparisons using bootstrap for the calculation of *p*-values. For several key outcomes, such as long-term side effects, adjusted age effects were calculated using logistic and linear regression and a fixed set of adjustment variables, which was defined based on clinical judgment. These variables were age, FIGO stage, surgery, surgical procedures (bowel resection, oophorectomy, hysterectomy, lymphonodectomy), chemotherapy, survival years, recurrent disease, and current disease status. *p*-values below 0.05 were considered statistically significant in an exploratory manner, indicating a signal of a potentially true effect.

## 3. Results

### 3.1. Patients’ Characteristics

Between November 2016 and November 2025, 1833 long-term survivors with ovarian cancer were recruited for the Expression VI study. Sixty-two patients were 18–40 years old at recruitment, compared to 1771 patients aged ≥41 years. Particularly when comparing summary statistics like the mean or percentages between groups, the reader should bear in mind the relatively small sample size in the group of young adult cancer survivors (YACS). The mean age at diagnosis in the YACS group was 25.0 years (range: 14–32 years) and 52.9 years (range: 10–88 years) in the patients’ group older than 41 years. Median survival time since initial cancer diagnosis was 11 years (range: 6–21 years) in the YACS group and 12 years (range: 6–49 years) in the older group.

There was no apparent difference in FIGO stages at diagnosis. However, 31.3% of the YACS group and 40.7% of the “older” group did not know their FIGO stage. Almost every patient had received primary surgery followed by chemotherapy (82.3% in the YACS group and 90.5% in the “older” group). There was no statistically significant difference in oophorectomies. However, hysterectomies were less frequently performed in the younger group (56.5% vs. 74.9%, *p* = 0.0016). In the subgroup of YACS with FIGO I Stage at the time of first diagnosis, the percentage of performed hysterectomy was even smaller (25%) in comparison with higher stages. There were no other differences in surgical procedures, as depicted in Table 1. The younger age group has lower recurrence rates—22.0% developed recurrent disease compared to 42.5% of the group above 41 years (*p* = 0.0274). At the time of recruitment, 10.7% in the YACS group and 14.0% in the group ≥41 years were receiving oncological treatment. Medication intake was more often reported in the older group; the medications reported were mainly beta-blockers (17.7% vs. 0%, *p* < 0.001), ACE-inhibitors (10.4% vs. 0%, *p* = 0.01), aspirin (8.3% vs. 0%, *p* = 0.0268), and statin (10.3% vs. 3.2%, *p* = 0.0834). According to the depicted medication, hypertension was found in 20.2% of the older group compared to 1.6% of the younger group (*p* < 0.001).

The younger cancer survivors have higher levels of education, with 39.3% having a university degree compared to 21.6% of the survivors older than 41 years (*p* < 0.001). Most of the YACS are working (86.9%), while only 27.7% of the older group work. In the older cohort, 61.1% are retired. More than one-third of the young patients do not have a partner (37.1% vs. 7.7%, *p* < 0.001). Three-quarters (76.8%) of the group ≥41 years have children compared to one-quarter (25.8%) in the YACS (*p* < 0.001), and 19.4% of the YACS still live in their parents’ household.

### 3.2. Health Status

No clear differences in reported health status were observed between the two cohorts. On a scale from 1 (very good) to 5 (very bad), the mean health status was 2.45 in the YACS vs. 2.51 in the older group. This observation remained after adjusting for the predefined set of covariables in a multiple linear regression (beta-coefficient 0.184; 95% confidence interval (CI): −0.372–0.741; *p* = 0.741). There was also no difference in the presence of physical symptoms in general. Half of both cohorts reported that they still had symptoms. Younger patients reported suffering more frequently from nausea and vomiting (44.4% vs. 25.2%; *p* = 0.01), bloating (59.3% vs. 49.9%, *p* = 0.038), weight loss (37% vs. 23.2%, *p* = 0.0302), constipation (60% vs. 45%, *p* = 0.0152), loss of appetite (40% vs. 24.9%, *p* = 0.0214), lymphedema (45.3% vs. 40.6%, *p* = 0.026), skin problems (14.5% vs. 5.3%, *p* = 0.072) and concentration difficulties (30.6% vs. 16.4%, *p* = 0.0048), while the older age group more frequently reported having polyneuropathy (20.3% vs. 4.3%, *p* = 0.002). YACS with bowel resection more frequently experienced gastrointestinal symptoms such as nausea, weight loss or loss of appetite.

In multivariate regression analyses adjusting for the previously mentioned set of covariates, YACS had a higher risk for gastrointestinal symptoms (OR 4.3; 95% CI: 1.44–12.48; *p* = 0.011), concentration difficulties (OR 4.98; 95% CI: 1.67–15.71; *p* = 0.005) and skin problems (OR 4.72; 95% CI: 1.24–15.31; *p* = 0.025).

**Figure 1:**
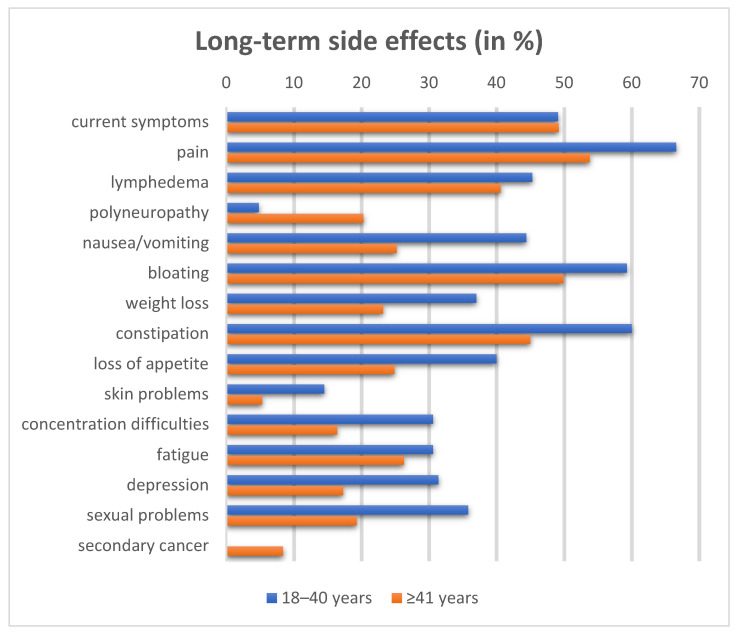
Long-term side effects according to age group (in %).

Fatigue was reported in 30.6% of the YACS group compared to 26.3% in the older group (*p* = 0.47) and was rated as one of the long-term side effects with the highest impact on quality of life (by 53.2% of the YACS and 38.2% of the group ≥41 years; *p* = 0.0236). Most support was given by friends and family (93.5% vs. 80.6%, *p* = 0.0124), the treating physician (33.9% vs. 36.5%, *p* = 0.689), the nurse (1.6% vs. 4.7%, *p* = 0.359), another patient (8.1% vs. 2.7%, *p* = 0.0284), or a self-help group (0% vs. 2.9%, *p* = 0.268). Long-term side effects are summarized in Figure 1.

Half of the younger patients still regard themselves as cancer patients. Younger patients showed a higher level of distress: the median distress on a scale from 0 (no distress) to 10 (maximum distress) measured with the distress thermometer was 6 (range 0–10) in the 18–40 years group and 4 (range 0–10) in the >41 years group (*p* = 0.0343). Differences in the items of the distress thermometer concerned insurance/finances (12.0% vs. 3.7%, *p* = 0.013), work/school (35.2% vs. 10.6%, *p* < 0.001), child care (11.5% vs. 3.3%, *p* = 0.0094), worries (61.1% vs. 44.7%, *p* = 0.019), sadness (56.1% vs. 37.2%, *p* = 0.0042), depression (31.4% vs. 17.3%, *p* = 0.0176), nutrition (26.9% vs. 11.2%, *p* = 0.0024), indigestion (48.1% vs. 27.7%, *p* = 0.002), dry nose (32.7% vs. 20.4%, *p* = 0.0418), polyneuropathy (28.8% vs. 42.9%, *p* = 0.0546) and sexual problems (35.8% vs. 19.3%, *p* = 0.0054).

In the ≥41 years group, 8.4% have had secondary cancer compared to 0% in the younger group (*p* = 0.0136). In both groups, 42% of patients had been genetically tested. BRCA mutations were found in 36.4% in the ≥41 years group compared to 3.8% (a single case) in the YACS (*p* = 0.0032), and 8.0% and 11.5%, respectively, did not know the result of their genetic testing. There was no clear difference in the percentage of patients having taken part in a clinical trial (*p* = 0.259).

Approximately 90.3% of YACS and 94.3% of the older group received follow-up care. Follow-up measures did not differ between the two groups. Of the woman older than 40, 53.9% received breast examination, such as a breast ultrasound or mammogram, compared to only 37.1% of YACS. Younger patients were less frequently involved in treatment decisions, and treatment alternatives were not offered to 72.9% of patients in the younger group (*p* = 0.006 and *p* = 0.331).

### 3.3. Lifestyle

**Figure 2:**
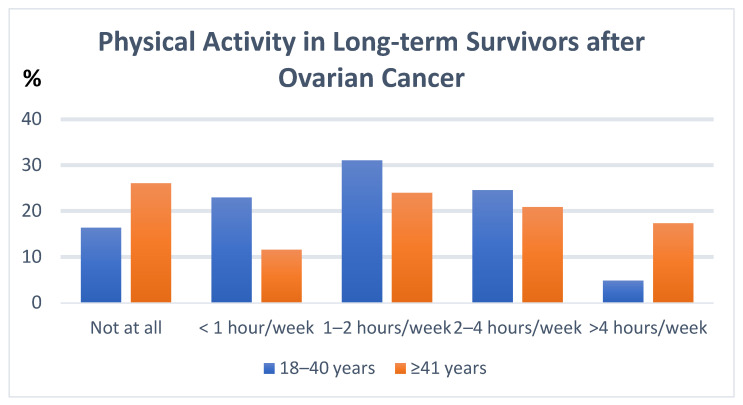
Physical activity of long-term survivors by age group.

Patients were also asked if they believed that there may be a reason for their cancer. Younger patients believed more frequently that contraceptives (14.5% vs. 3.5%, *p* < 0.001), environmental factors (38.7% vs. 17.2%, *p* < 0.001) and nutritional factors (16.1% vs. 6.7%, *p* = 0.01) were reasons for the development of their cancer diagnosis. YACS were physically more active (*p* = 0.0028) (see Figure 2). More than one-third of the younger group is exercising more often than before their cancer diagnosis, and 45.8% believe that regular physical activity positively influences their cancer journey.

In contrast, smoking was more evident in the younger group, with 19.7% vs. 10.9% still smoking after cancer diagnosis. Consumption of 2–3 alcoholic drinks per week was reported by 12.5% of the patients above 41 compared to 3.2% of those in the younger group, and 2–4 alcoholic drinks per month were more frequently reported by the younger group (35.5% vs. 22.6%, *p* = 0.0536). Overall, 61.3% of the younger patients have changed their nutrition habits after diagnosis, and 62.9% believe that nutrition has a positive impact on the course of their disease. Additionally, 87.2% of the younger patients are interested in studies about long-term cancer survivorship compared to 68.8% of the older group (*p* = 0.0174).

## 4. Discussion

The increasing incidence of cancer among young adults is a very concerning matter, and there is an urgent need for more specific research among this unique group of patients to adjust the follow-up care to their needs. By evaluating the subgroup of young adults from the Expression VI study, which is focused on long-term ovarian cancer survivors, we provide valuable insights into this subgroup of patients. In this exploratory analysis, we found signs of potentially significant differences in the burden of different side effects between younger and older ovarian cancer survivors. Gastrointestinal symptoms, concentration difficulties, and distress appeared to be higher in the young adult cancer survivor group, while polyneuropathy and secondary cancers were more often reported in the group >41 years.

Given the exploratory nature of the analyses and the large number of comparisons performed, *p*-values should be interpreted with care, and findings should be considered hypothesis-generating. Also, the question of the etiology of rising cancer incidences may be multifactorial. Changes in testing availability, lifestyle, genetic factors, and environmental exposures are considered fundamental factors [2]. The presence of gene alterations in the BRCA 1 and 2 genes, and a positive family history are known risk factors for ovarian and breast cancer. Nowadays, BRCA testing is routinely offered to ovarian cancer patients. Many long-term survivors, however, have been diagnosed before the implementation of BRCA testing into clinical routine, so that many LTS do not know their mutation status. However, BRCA mutations are not only associated with ovarian cancer but also with breast cancer and also bear a risk for family members. LTS should therefore be counseled about BRCA testing, intensified screening programs and preventive surgery. The history of BRCA testing was very low in our cohort and even lower in the YACS. In addition to the already-mentioned reason that many LTS have been diagnosed before routine BRCA screening for ovarian cancer, other reasons could be that patients do not know the results or are not sure if they were tested. Low-grade ovarian cancer is more frequent in younger patients and is not associated with BRCA, so it may be assumed that those might not have been tested. However, within the Expression VI survey, we did not ask patients for their histological subtype, as from our clinical experience, they are unlikely to know their subtype. In the present study, only one-third of the patients associated their diagnosis with the presence of cancer in the family. The most significant factors for developing the cancer from the perspective of young adult cancer survivors (YACS) in the current study were stress in the family (35.5%), diet (16.1%), environmental influences (38.7%), and the usage of contraception (14.5%).

### 4.1. Long-Term Side Effects

The impact of cancer on fertility and impairment of ovarian function due to cancer treatment is a major concern of YACS and may increase the psychological burden. Consultation for fertility preservation and family planning is of great importance, and discussion of fertility alteration due to cancer treatment should be performed multiple times during the patient journey [24].

In 95.2% of the younger patients in this population, the ovaries were removed during the primary surgery, but a hysterectomy was less often performed (56.6%) in comparison to older patients. The current study did not collect information on whether fertility preservation consultation was performed, but due to the significantly higher number of young patients who preserved their uterus after the primary surgery, we suggest that their family planning was likely not completed and that the relevance of consultation on fertility options after the end of treatment is high among YACS.

Further aspects to be considered are changes in sexual health. Between 30% and 100% of cancer survivors experience sexual dysfunction, with an average of 50–60%; this is especially frequent among gynecologic cancer survivors (78%), as discussed by Cherven et al. [15]. In the current study, 35.8% of YACS experienced sexual problems, compared to 19.3% of the older women. Cancer survivors may have barriers to discussing sexual health issues, but also some medical oncologists and gynecologists are not sufficiently trained. In such cases, actively addressing sexual problems in the follow-up consultation could help, along with, if necessary, referral to a specialized consultation with a sexual medicine specialist.

The YACS experienced more frequent symptoms of cognitive impairment, such as memory difficulties, and had psychological distress, such as anxiety, depression, and sadness. They were also more concerned about socioeconomic aspects such as work, education, child care, and insurance/finances. According to the literature, up to 30% of YACS have problems in completing higher education and maintaining full-time employment [16,18]. Monitoring YACS for psychological and neurocognitive concerns is of great importance to provide specific psychological intervention for emotional distress for those at risk and thus facilitate the adaptation and reentry to school and work.

A cross-sectional study compared symptom burden between AYA (adolescents and young adults 18–39) and a group of older adults ≥40 years and reported that AYA had higher rates of moderate (26.1% vs. 18.9%) and severe (10.7% vs. 7.7%) anxiety (*p* < 0.001) and moderate (20.8% vs. 17.1%) and severe (7.4% vs. 6.5%) depression (*p* < 0.001) compared with older adults with cancer [25]. In accordance with the previous study, the YACS in our cohort had a higher level of distress and depression symptoms (*p* < 0.001).

A consultation with a social worker and psychooncological support should be provided during and after the treatment to address these specific needs. Interestingly, despite having a higher education status than the elder group, the YACS group was less frequently involved in treatment decisions. We can only speculate, but reasons might be a higher psychooncological burden or less life experience than the older cancer patients.

Similar to knowledge from other tumors, such as colorectal cancer [26], YACS with ovarian cancer had more frequent gastrointestinal symptoms, such as nausea, vomiting, lack of appetite, and weight loss. Gastrointestinal symptoms remained a significantly more frequent side effect after multivariate analyses. Young age is actually known to be a risk factor for chemotherapy-induced nausea and vomiting. This association needs further exploration. In the multivariate analysis, the YACS had a higher risk for concentration difficulties and skin problems.

Comparable data are published in two cancer register studies. In the NOR-CAYACS study from the Cancer Registry of Norway, among childhood, adolescent, and young adult cancer survivors, 61.5% reported the experience of at least one late effect, with the most common being concentration/memory problems (28.1%) and fatigue (25.2%) [27]. In a Netherlands Cancer Registry study, AYA breast cancer survivors had a higher risk of long-term health challenges such as musculoskeletal, psychological/psychiatric, skin, and urinary tract conditions compared to age-matched controls [28].

These long-term problems and symptoms should be actively addressed in the cancer surveillance programs.

### 4.2. Lifestyle

In the YACS cohort, more women consume alcohol and nicotine. According to data from the USA, young adult cancer survivors (aged 18–44) reported the highest rates of current cigarette smoking (27.9%) and current e-cigarette use (11.8%) [29].

Similar results from a USA state-based random-digit-dial telephone surveillance program demonstrated that AYA cancer survivors, compared with respondents who had no history of cancer, reported a significantly higher prevalence of current smoking (26% vs. 18%); obesity (31% vs. 27%); chronic conditions, including cardiovascular disease (14% vs. 7%), hypertension (35% vs. 29%), asthma (15% vs. 8%); disability (36% vs. 18%); and poor mental health (20% vs. 10%) and physical health (24% vs. 10%) [30].

Promoting healthy habits, such as reduction of alcohol consumption, smoking cessation, and increasing exercise, is of great importance to decrease the risk of late effects such as cardiovascular diseases and second malignancies.

Adherence to vigorous-intensity exercise guidelines was associated with a 51% reduction in the risk of any cardiovascular event in comparison with not meeting the guidelines (*p* = 0.002) among a cohort of YACS with Hodgkin lymphoma [31].

The YACS under the age of 40 in the present study were more physically active, and one-third changed their exercise activity after the diagnosis, which shows a high motivation for a healthy lifestyle.

More education and information about the importance of lifestyle changes should be provided in the cancer surveillance programs for YACS. Certain factors, such as physical limitations, fatigue, cancer type, and socioeconomic status, can limit the willingness to participate in physical activity (PA), while others, such as including families and friends, connecting survivors, and providing social support together with increasing motivation, are key strategies for the promotion of physical activity in young cancer survivors [32]. A qualitative study explored several factors regarding tailoring physical activity programs to YACS and identified two major factors: developing interventions that are convenient and that provide social support [33].

### 4.3. Secondary Cancer Prevention

YACS in high-income countries are shown to have a 1.3- to 2-fold higher risk of developing any secondary cancer in comparison to the general population, with the risk increasing over time to 10.3% in females at 25 years’ follow-up [34]. Screening for breast cancer is available in Germany starting at the age of 50, except for the high-risk population who are carriers of a genetic mutation and who follow a more intensified and earlier screening. Beginning breast cancer screening at an earlier age, using modalities such as mammography or mammasonography, should be considered due to the higher risk of second malignancies in the YACS population. Secondary cancer in the group of >40 years was 8.4%, which is within the range reported in the literature. There were no secondary cancers in the YACS group in our cohort. Cancer incidence rates increase with increasing age, which might be the reason for the higher rates in the older group. Furthermore, there was a reported BRCA mutation rate of 36% in the older group. Especially in this high-risk group, after ovarian cancer, breast examinations and mammograms should be more frequently performed, as reported in our cohort (53.9% in the older group and 37.1% in the YACS group). Furthermore, in survivorship care, the risk for secondary cancer should be addressed, and patients should be motivated to take part in cancer screening programs.

### 4.4. Strengths and Limitations

A major strength of the current study is that it is a multicenter international survey focused on long-term side effects, perspectives, and expectations of the growing patient cohort of long-term survivors of ovarian cancer. To our knowledge, this is the largest cohort of long-term cancer survivors with epithelial ovarian cancer worldwide. In evaluating a cohort of young adult cancer survivors, we examined long-term health issues and practical problems, providing both healthcare providers and survivors with important insights to improve health outcomes and adapt cancer surveillance programs in the future. Another strength is that not only university hospitals but also non-university hospitals and practices could recruit patients, reflecting the broad range of medical contact points of long-term survivors. The recruitment via advocacy groups and patient conferences stands within patient empowerment and active patient involvement in studies.

An important limitation of the current study is that it is based on self-reported patients characteristics and problems, and due to the small number of YACS, the results may not be generalized for a larger population. Ovarian cancer is usually a cancer diagnosis of postmenopausal women, making it difficult to gain a large cohort of young adult cancer survivors after ovarian cancer. Therefore, large international multicenter studies are warranted for this rare patient subgroup of YACS with ovarian cancer. Nevertheless, it is the largest existing cohort of long-term ovarian cancer survivors. With the growing population of YACS, more studies addressing their specific problems and symptoms and tailored cancer survivorship programs for YACS with ovarian cancer are needed.

## 5. Conclusions

The current study summarized self-reported long-term treatment-related physical symptoms, psychological issues, and concerns in practical aspects of daily life, such as child care, work, and education, for one of the largest cohorts with long-term survivors of ovarian cancer. Half of the YACS face multiple challenges and experienced cancer-related symptoms even after the end of cancer treatment. Gastrointestinal symptoms, concentration difficulties, and distress are more frequent in the YACS, while polyneuropathy is more frequently found in the elder group. In order to meet their needs, cancer surveillance programs should be tailored and include multidisciplinary teams, with the possibility for specific consultation on topics such as fertility preservation, sexuality, psychological and social/practical support during and after cancer therapy. Genetic testing rates were very low, and genetics should be routinely addressed in survivorship care. Future studies should be focused on developing strategies for the early detection of long-term health problems to reduce disability, loss of work and education opportunities and improve quality of life in the group of YACS. Interventional studies are especially needed to evaluate whether multidisciplinary survivorship programs addressing the needs of our patients can improve physical and mental health status as well as quality of life.

## Figures and Tables

**Table 1:** Patients’ characteristics.

		Whole Cohort (n = 1833)	18–40 Years (n = 62)	≥41 Years (n = 1771)	*p*
Median survival time (years)		12 (range: 6–49)	11 (range: 6–21)	12 (range: 6–49)	
FIGO	I	217 (16.5%)	12 (22.2%)	205 (16.2%)	0.17
	II	138 (10.5%)	3 (5.6%)	135 (10.7%)	
	III	452 (34.3%)	16 (29.6%)	436 (34.5%)	
	IV	92 (7.0%)	1 (1.9%)	91 (7.2%)	
	Not known	417 (31.7%)	22 (40.7%)	395 (31.3%)	
Recurrent disease	Yes	752 (41.9%)	13 (22.0%)	739 (42.5%)	0.0274
Primary surgery	Yes	1797 (99.0%)	61 (100%)	1736 (98.9%)	0.659
Oophorectomy	Yes	1678 (91.5%)	59 (95.2%)	1619 (91.4%)	0.368
Hysterectomy	Yes	1362 (74.3%)	35 (56.5%)	1327 (74.9%)	0.0016
Omentectomy	Yes	833 (45.4%)	27 (43.5%)	806 (45.5%)	0.787
Lymphonodectomy	Yes	970 (52.9%)	35 (56.5%)	935 (52.8%)	0.615
Bowel resection	Yes	243 (13.3%)	9 (14.5%)	234 (13.2%)	0.856
Chemotherapy	Yes	1641 (90.3%)	51 (82.3%)	1590 (90.5%)	0.093
Marital status	Single	157 (8.8%)	23 (37.1%)	134 (7.7%)	<0.001
Children	yes	1342 (75.1%)	16 (25.8%)	1326 (76.8%)	<0.001
University degree	yes	390 (22.2%)	24 (39.3%)	366 (21.6%)	<0.001

## Data Availability

The data are not yet publicly available due to the fact that the study is still recruiting.

## Abbreviations

The following abbreviations are used in this manuscript:

YACS	Young adult cancer survivor
LTS	Long-term survivor
